# One-step growth of multilayer-graphene hollow nanospheres via the self-elimination of SiC nuclei templates

**DOI:** 10.1038/s41598-017-13143-3

**Published:** 2017-10-23

**Authors:** Byeong Geun Kim, Deok-Hui Nam, Seong-Min Jeong, Myung-Hyun Lee, Won-Seon Seo, Soon-Mok Choi

**Affiliations:** 1 School of Energy, Materials and Chemical Engineering, Korea University of Technology and Education, Cheonan, 31253 Korea; 2 Energy & Environmental Division, Korea Institute of Ceramic Engineering and Technology, Jinju, 52851 Korea

## Abstract

We introduce a one-step growth method for producing multilayer-graphene hollow nanospheres via a high-temperature chemical vapor deposition process using tetramethylsilane as an organic precursor. When the SiC nuclei were grown under an excess carbon atmosphere, they were surrounded via desorption of the hydrocarbon gas species, and graphene layers formed on the surface of the SiC nuclei via the rearrangement of solid carbon during the heating and cooling. The core SiC nuclei were spontaneously removed by the subsequent thermal decomposition, which also supplied the carbon for the graphene layers. Hence, multilayer-graphene hollow nanospheres were acquired via a one-step process, which was simply controlled by the growth temperature. In this growth process, the SiC nuclei acted as both the template and carbon source for the formation of multilayer-graphene hollow nanospheres.

## Introduction

Over the past few decades, research on carbon nanostructures has been extensively undertaken in various fields, such as electronic devices and energy systems- and bio-systems. Divserse shapes have also been developed, e.g., carbon nanotubes (CNTs)^1^, graphene^2^, carbon anions^3–6^, graphene oxides^7,8^, and carbon nanospheres filled with and without metal nanoparticles^9–17^. Moreover, complexes with carbon nanostructures have been used in applications such as Li-ion batteries^18^, supercapacitors^19–21^, photovoltaic devices^22^, thermal energy storage^23^, solar cells^24^, and thermoelectric devices^25,26^.

In particular, hollow carbon nanospheres have been utilized owing to their excellent performance, large reversible capacity, and good cyclability. For supercapacitors^27^ and Li-S batteries^28^, the large specific surface area and short diffusion distance of hollow carbon nanospheres can be advantages. Methods such as electron-, laser-, and chlorine-assisted methods have been developed to produce hollow carbon nanospheres^11,13,27^. However, the electron- and laser-assisted methods are not suitable for the mass production of graphitic carbon nanospheres^11–13^. The choline-assisted method has mainly been used to fabricate graphitic carbon nanospheres from carbides, but follow-up processes are needed to remove residual chlorine after the chlorine treatment^27,29^. Hence, an easier mass production method to produce high-quality hollow carbon nanospheres is essential for their future industrial feasibility.

Carbon nanostructures have been produced from various carbides, such as SiC^18,30–32^, TiC^27^, and Ti_2_AlC^29^, via the selective extraction of metal from carbides using chlorination and thermal treatments. Cambaz *et al*. reported the formation of a carbide-derived carbon on beta-SiC whiskers via heat treatment under a high vacuum atmosphere^18^. They observed that all the SiC whiskers completely transformed into graphitic whiskers above 2000 °C. Graphene^30^ and CNTs^31,32^ were also grown on carbides via the thermal decomposition of carbides and out-diffusion of carbon. These studies indicated that the carbides act as both a template and source for fabricating carbon nanostructures^27–32^. In addition, hydrocarbon gas species can be used as carbon sources, and a high temperature can promote the graphitization of the carbon nanostructures^17^. From the results of the previous research, the important factors for the production of graphitic carbon nanostructures are summarized as follows: (1) carbide templates, (2) thermal treatment at high temperatures, and 3) a hydrocarbon supply.

In consideration of these requirements, a high-temperature chemical vapor deposition (HTCVD) process could be a solution for the mass-production of graphitic carbon nanostructures. We previously reported the growth of 6H-SiC single crystals with a tetramethylsilane (TMS, Si(CH_2_)_4_) precursor using a HTCVD^33^. Commonly, HTCVD has been used to grow crystalline bulk materials, mainly SiC ingots, with a wide band gap and a high melting temperature^33–38^. Ellison *et al*. explained the growth mechanism of SiC crystals using HTCVD as follows^35^. When the precursors are injected into the chamber of the HTCVD system with a carrier gas (H_2_), they are thermally decomposed as the temperature increases. Passage through the ‘heat zone’ of the HTCVD system induces the formation of Si_x_C_y_ clusters. When the Si_x_C_y_ clusters reach the surface of the SiC seed crystals in the ‘growth zone’, they are adequate sources for growing the SiC crystals (Fig. S1). In a HTCVD process, mass production is possible because the sources, such as the gas species, are continuously supplied to the HTCVD chamber during the growth process. Moreover, the ratio of C to Si can be easily controlled via the flow of the source and carrier gases and the design of susceptors^33,37,38^. This aspect is important because controlling the Si/C ratio determines whether SiC single crystals can be obtained without excess carbon. Finally, the operating temperature is very high (approximately 2000 °C) and suitable for growing highly ordered crystalline materials. For these reasons, we are convinced that the unique characteristics of the HTCVD process create a favorable environment for the formation of graphitic carbon nanostructures.

In this study, we successfully demonstrated the growth of multilayer-graphene hollow nanospheres (MHNs) using a TMS-based HTCVD process. When a conventional CVD process was utilized for the fabrication of carbon nanospheres in previous reports, solid templates were separately prepared, and additional steps, such as the removal of the templates, were needed to obtain pure carbon nanospheres^39,40^. In a TMS-based HTCVD process, however, the production of MHNs is simply controlled by the growth temperature. In addition, the template materials (SiC nuclei) can be continuously produced and spontaneously removed after the fabrication of the MHNs due to the high process temperature. This strategy facilitates a one-step process to produce MHNs without any follow-up steps. The comprehensive growth mechanisms of the MHNs are discussed with the experimental results and thermodynamic calculations herein.

## Results and Discussion

**Figure 1:**
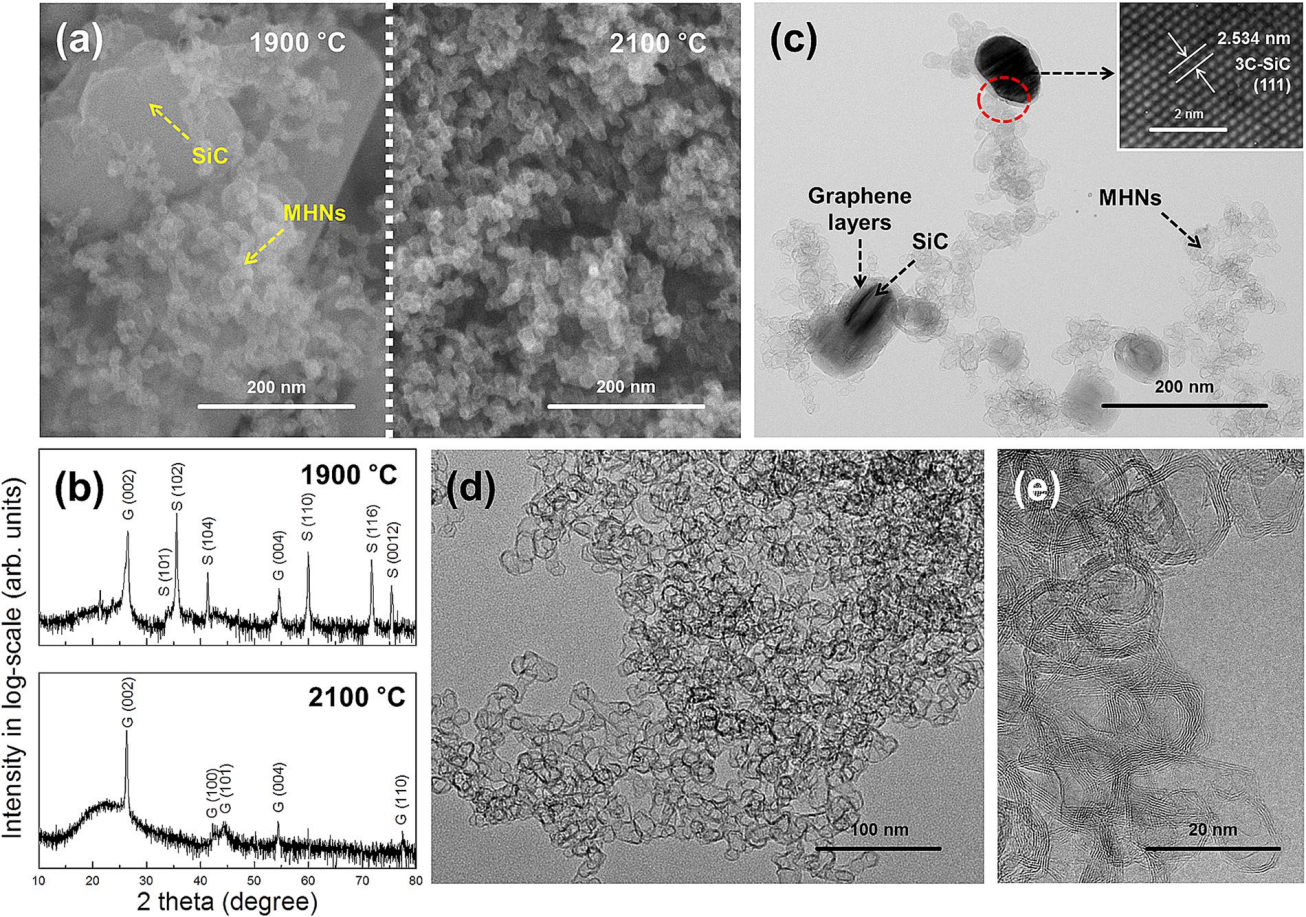
(**a**) FESEM images and (**b**) XRD patterns of the products acquired at different temperatures (1900 °C and 2100 °C). S and G in the XRD patterns denote the SiC and graphite phases, respectively. TEM images of the product obtained at (**c**) 1900 °C and (**d**,**e**) 2100 °C. Inset in Fig. 1(c) is the HRTEM image of the SiC crystal.

Figure 1(a) presents the FESEM images of the products acquired at 1900 °C and 2100 °C. The SiC crystals and MHNs were easily distinguished by the small size of the MHNs (diameter <20 nm). Moreover, the FESEM images show the circular shape of the MHNs. In the XRD analyses (Fig. 1(b)), the SiC and graphite phases were found in the products acquired at 1900 °C, but only the graphite phases were detected at 2100 °C. All the peaks were indexed with 3C-SiC (JCPDS no. 29–1131) and graphite (JCPDS no. 41–1487). In the case of the product obtained at 1900 °C, the corresponding peaks for 6H-SiC and 3C-SiC were found in the Raman analysis (Fig. S2). In the FESEM- and TEM-EDS analyses, only C was detected in the product obtained at 2100 °C, whereas both Si and C were found in the product obtained at 1900 °C (Figs S3–S4). These results indicated that both SiC and graphite formed at 1900 °C whereas only graphite formed at 2100 °C.

**Figure 2:**
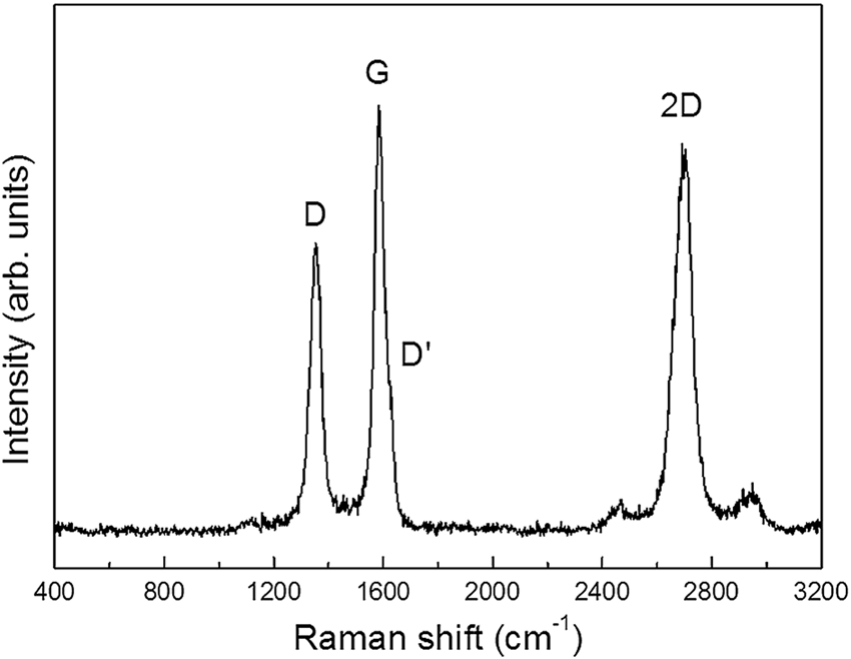
Raman spectrum of the MHNs obtained at 2100 °C.

Figure 1(c) the shows TEM image of the products acquired at 1900 °C. A high-resolution TEM (HRTEM) image of the SiC crystal clearly shows the single-crystalline nature of 3C-SiC (inset of Fig. 1(c)). Notably, the SiC crystals were covered with graphene layers. We will explore this aspect later in detail. Figure 1(d) and (e) show the TEM images of the products obtained at 2100 °C. Only the MHNs were observed, and they were composed of highly oriented graphene multilayers with empty interiors. The value of the d-spacing between the graphene layers was 0.365 nm, which was slightly larger than that of ideal graphite (0.335 nm)^3^. The additional TEM images of the products obtained at 1900 °C and 2100 °C are shown in Fig. S5. Figure 2 shows the Raman spectrum of the MHNs acquired at 2100 °C. The peaks at ~1354.5 cm^−1^, ~1624 cm^−1^, ~1585.5 cm^−1^, and ~2700 cm^−1^ represent the defect-induced peaks (D and D’), crystalline graphite peak (G) and second-order peak (2D), respectively, of graphite^41,42^. The I_D_/I_G_ ratio was 0.79 ± 0.02. No SiC or Si peaks were found, which was consistent with the results of the EDS and XRD analyses (Figs 1b and S3–S5). The results of the XRD, FESEM, and Raman analyses were also consistent with those from the XPS (Fig. S6). Finally, we confirmed that the formation of the MHNs and SiC crystals was governed by the growth temperature. MHNs without SiC crystals could be produced at temperature over 2100 °C.

**Figure 3:**
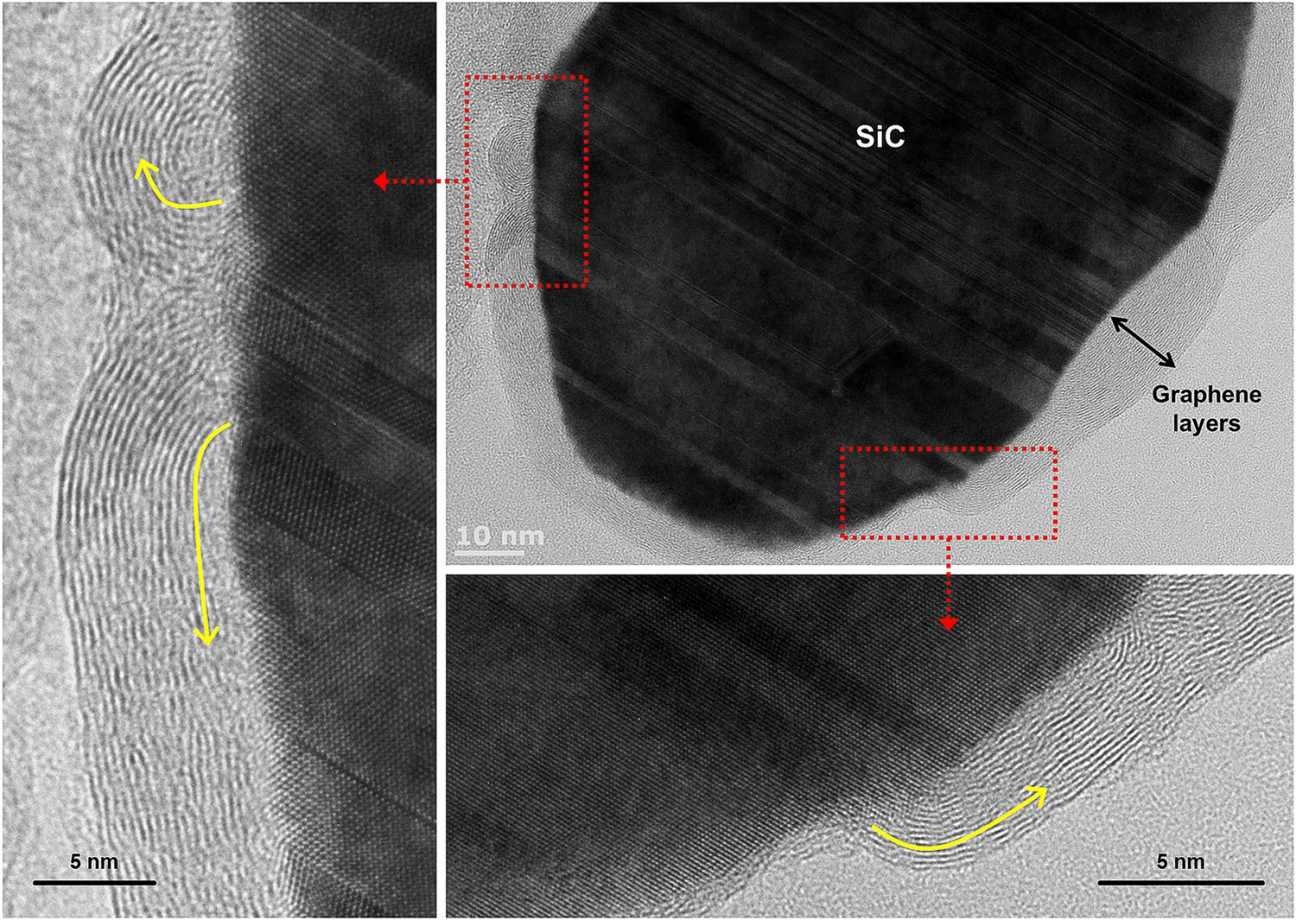
(**a**–**c**) TEM images of the SiC crystal obtained at 1900 °C, which is shown in the red dotted circle in Fig. 1(c).

**Figure 4:**
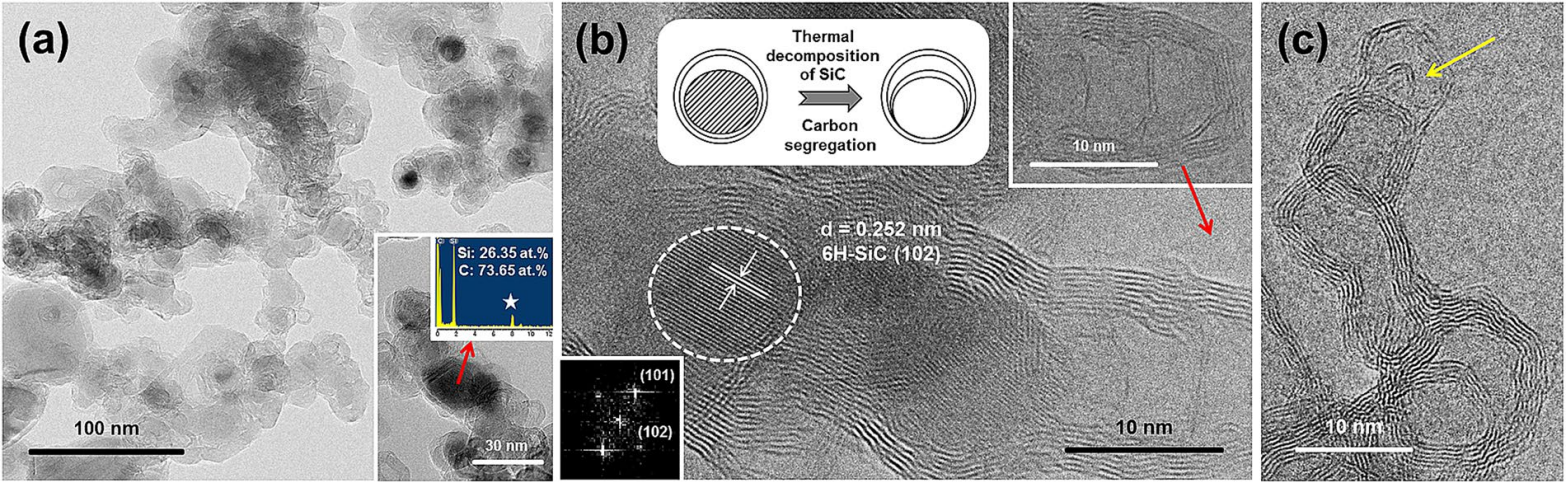
(**a**,**b**) TEM images and EDS spectrum (inset of Fig. 4(a)) of the product acquired at 1500 °C. The white asterisk in the EDS spectrum indicates the peak of the Cu grid. (**c**) TEM image of MHN-2100.

Figure 3 shows the enlarged TEM images of the surface between the graphene layers and the SiC crystal, which is indicated by the red dotted circle in Fig. 1(c). The TEM images clearly indicated that the SiC crystal is surrounded by graphene layers. The HRTEM images of the red dotted squares in Fig. 3 show that the growth of the graphene layers started perpendicular to the surface of the SiC crystal (yellow arrows). This graphene growth behavior is similar to that reported in previous studies of the direct growth of graphene layers from SiC crystals. Recently, many research groups have demonstrated that graphene layers are directly grown via the thermal decomposition of bulk SiC at temperatures 1225 °C~2000 °C without additional sources in a vacuum or Ar gas atmosphere^30,43,44^. The TEM analyses shown in Fig. 3 indicate that the C source for the graphene layers originated from the SiC crystals. A similar growth behavior was also observed at a low temperature (1500 °C), as shown in Fig. 4.

Figure 4(a) and (b) show the TEM analyses of the products obtained at 1500 °C. In this study, the MHNs obtained at different temperature were named ‘MHNs-temperature’ (for example, MHNs-1500). We found that some MHNs-1500 were not hollow, which was identified by the different contrast in the TEM images (Fig. 4(a) and Fig. S7). The TEM-EDS analysis (inset in Fig. 4(a)), HRTEM images (Fig. 4(b)), and fast Fourier transform (FFT) (bottom and left inset of Fig. 4(b)) images show that SiC crystals with a small size (<15 nm) filled the interior of the MHNs-1500, which was not observed in the MHNs-2100. The small SiC crystals embedded in the MHNs were named ‘SiC nuclei’ to separate them from the SiC crystals. The HRTEM image (white dotted circle in Fig. 4(b)) clearly shows the (102) plane with the d-spacing (0.252 nm) of 6H-SiC. Two bright dots in the FFT image represent the (101) and (102) planes of 6H-SiC. The results of the TEM analyses are further shown in Fig. S8.

We observed a unique growth form of the MHNs (top and right inset of Fig. 4(b) and (c)), which resembles bamboo. This growth form has been observed for the growth of CNTs with metal catalysts, such as Ni nanoparticles^45^. When metal catalysts gradually decompose at high temperatures, the segregation or bulk diffusion of C atoms can form graphene layers^45^. If the surface of the SiC nuclei thermally decomposes, e.g., in our case, the Si atoms can be removed, and graphene layers can form via self-organization of the remaining C atoms. Hence, the volume of the SiC nuclei gradually decreases, and the bamboo-like growth can form by via the subsequent sublimation of the SiC nuclei (top and middle insets in Fig. 4(b,c), respectively)^10,45^. From the results of TEM analyses (Figs 3–4), we confirmed that the growth of the graphene layers via the thermal decomposition of SiC provides important information about the growth mechanism of the MHNs. The SiC nuclei can act as templates and sources for the growth of the MHNs.

**Figure 5:**
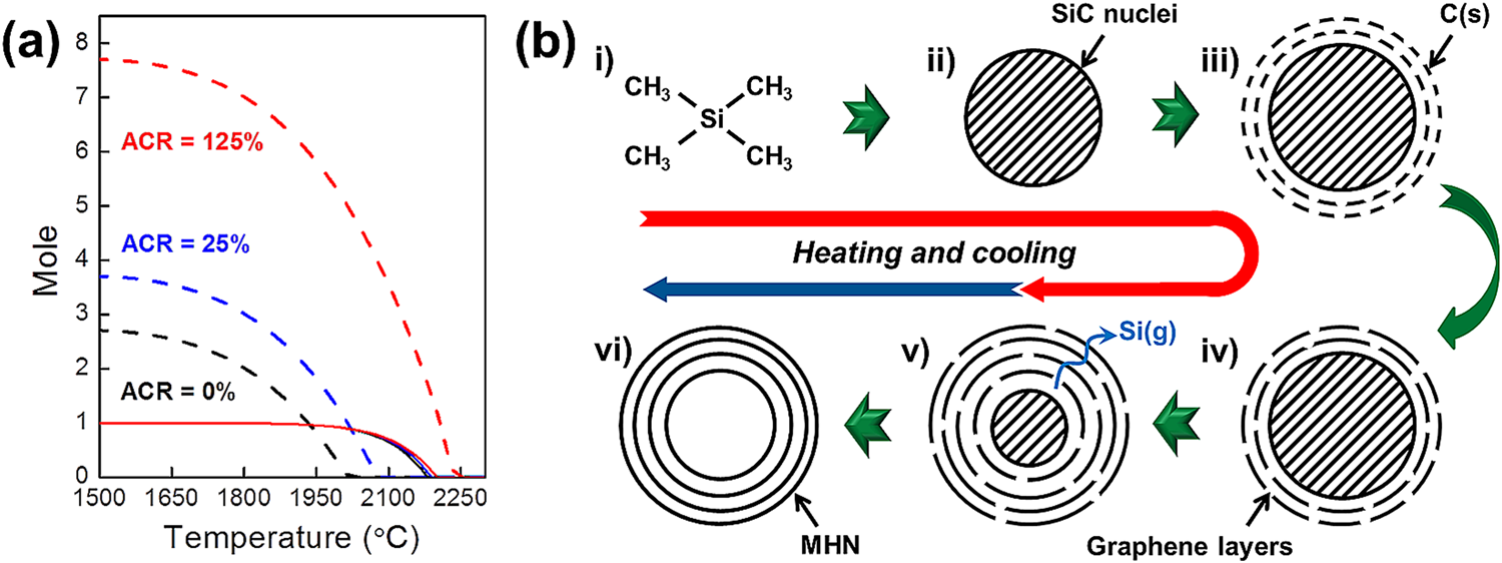
(**a**) Thermodynamic equilibria with the formed solids as a function of temperatures. Solid and dotted lines indicate the moles of SiC(s) and C(s), respectively. (**b**) Schematic diagram showing the growth mechanisms of MHNs in in the HTCVD process (heating and cooling time): i) Injection of TMS with the H_2_ carrier gas, ii) the formation of SiC(s) (SiC nuclei), iii) the formation of C(s) on the SiC nuclei, iv) the formation of graphene layers via the rearrangement of C(s), v) the evaporation of Si and decomposition of the SiC nuclei, and vi) the formation of the MHNs.

It should be noted, however, that other sources (hydrocarbon gas species) were supplied during the TMS-based HTCVD process. This method is different from previous reports about the direct growth of graphene layers on SiC^30,43,44^. There are two methods to grow graphene layers on SiC: 1) direct growth via the thermal decomposition of SiC without the supply of other C sources^30,43,44^ and 2) epitaxial growth with the supply of hydrocarbon gases such as propane^46^. In the former case, the growth of the graphene layers is performed under a vacuum or under an Ar gas atmosphere, and no external sources are supplied except SiC^30,43,44^. In the latter case, hydrocarbon gas species, such as CH_4_ and C_2_H_2_ are supplied as the sources for the graphene layers^17,46^. Considering our experimental conditions (extremely high temperature and the supply of C-containing gas species), the growth from the desorption of hydrocarbon species must be considered along with the direct growth from SiC because 27 gas species, such as CH_4_, C_2_H_2_, H, Si, and Si_2_C, are formed in the ‘growth zone’ during the TMS-based HTCVD (Fig. S9). Thus, we investigated the influence of the gas species on the growth of the graphene layers and SiC crystals using classical thermodynamic calculations (Fig. 5(a)).

The thermodynamic calculations were performed using the same parameters (working pressure, temperature, and starting materials) as the experimental conditions. Based on the Gibbs energy of formation of the compounds (pure substances) at the equilibrium state, the moles of the products (solids and gases) were obtained as a function of the temperature using a thermodynamic program (FactSage^TM^) as a function of the temperature. In the HTCVD system, the abnormal fluid flow of the unreacted gas species can occur near the outlet, which is called a ‘bottleneck’ phenomenon^38,47^. This effect occurs due to the accumulation of carbon in the ‘growth zone’ via the condensation of unreacted gas species near the outlet. As a result, the formation of C(s) at high temperatures is enhanced, which was verified by the thermodynamic calculations^38^ and the mass/heat transfer analysis by the finite element method (FEM)^47^. As carbon accumulates over time, TMS is directly injected into the carbon-accumulated atmosphere. Hence, the amount of carbon in the thermodynamic modeling can be estimated as the increasing carbon ratio per TMS molecule (ACR), *ACR* = *(the amount of the additional carbon/the amount of carbon in a TMS molecule)* × *100* ^40^. In consideration of this factor, the thermodynamic equilibria of the solids as a function of temperature are shown in Fig. 5(a). When the temperature increased, the formed content (mole) of C(s) and SiC(s) gradually decreased (Fig. 5(a)). The moles of C(s) gradually decreased above 1500 °C, whereas that of SiC(s) decreased above 2000 °C. However, the moles of C(s) were much higher than that of SiC(s) with the increase in the ACR.

Based on the results of the experiments and the thermodynamic simulations, we propose a possible growth mechanism for the MHNs, as shown in Figs 5(b) and S10. When the TMS precursors are injected into the quartz tube for the HTCVD, they are thermally decomposed into Si- and C-containing gas species. If no bottleneck phenomenon occurs under the experimental conditions for obtaining only SiC(s), the SiC nuclei form via the chemical reaction between the Si- and C-containing gas species under the thermodynamic equilibrium state. They grow into SiC crystals via both the continuous supply of the sources and coalescence with other SiC nuclei. Hence, high-quality SiC crystals can be grown.

However, the growth of the SiC nuclei can be disturbed by excess C(s). When C(s) is deposited on the surface of the SiC nuclei, it forms graphene layers, and the growth of the SiC nuclei may be limited by the layers because the formed-graphene layers act as passivation layers to prevent the supply of source molecules with a large size^32^. As a result, the core SiC nuclei embedded in the MHNs may begin thermally decomposing due to the high thermal energy. When SiC(s) is stably formed at 1500 °C~2000 °C (Fig. 5(a)), some of the SiC nuclei can grow into large SiC crystals. However, this growth is difficult at 2100 °C because the formation of SiC(s) is unstable above 2000 °C (Fig. 5(a)). For this reason, no SiC crystals were found in the products obtained at 2100 °C.

When the growth process ends, the heating and supply of the TMS are stopped. At this time, only the vacuum was maintained as the temperature slowly cooled to room temperature over 2 hours. Until this atmosphere, it is possible for thermal decomposition of SiC to occur during the cooling because the thermal decomposition of bulk SiC occurs at low temperatures (~1500 °C) and in a short time (30 min)^43,44^. After the growth, well-organized graphene layers formed on the surface of the SiC crystals (Fig. 3). In the case of the graphene-encapsulated SiC nuclei, their thermal decomposition may be accelerated by the ‘size effect’. This aspect is a unique characteristic of nanostructures compared with that of bulk materials,^48–53^ and the thermal characteristics of these materials are dependent on their size^49^. For example, the melting temperatures of Sb_2_Te_3_ and GeTe nanowires are two-thirds lower than those of the bulk materials^52,53^. In addition, the enthalpy of fusion decreases with the decreasing size of materials^49^. Si may evaporate and pass between the graphene layers during the thermal decomposition of SiC^10^. Hence, nanoscale SiC in graphene-encapsulated SiC nuclei can be completely removed during cooling after heating at high temperatures (at 1900 °C and 2100 °C). Therefore, this result can explain why the experimental results and thermodynamic calculations were different from each other at 2100 °C. Although the moles of C(s) and SiC(s) abruptly decreased with the increase in temperature, the results of the thermodynamic calculations indicated that they still formed at 1500 °C~2100 °C. However, only C(s) remained at 2100 °C in the real experiment (Fig. 1). To explain the reason for the discrepancy, we note that the thermodynamic calculations only show the reactions at the corresponding temperatures. In a real experiment, however, the entire growth process was divided into two steps (heating and cooling) as a function of the temperatures. The reactions during cooling are not considered by the thermodynamic calculations (Fig. 5(a)). At low temperatures (below 1500 °C), the complete removal of the core SiC nuclei may not occur due to the insufficient thermal energy, even if the reaction in the cooling step was considered. Thus, traces of the SiC nuclei remained in the MHNs-1500 (Fig. 4(b)).

We believe that this simple and efficient method can be used for the mass-production of graphitic carbon nanospheres for various applications, such as supercapacitors, gas sensors, energy storage and conversion, and biomedical systems, and contribute to a better understanding of the fabrication mechanisms and crystal growth behaviors of carbon nanostructures.

## Conclusions

In summary, we successfully demonstrated a one-step growth of MHNs using a TMS-based HTCVD process. When the TMS precursors were injected into the heating zone of the HTCVD, SiC nuclei formed via the chemical reaction between the Si- and C-containing species. When an excess carbon atmosphere was reached in the growth zone, solid C was deposited on the surface of the SiC nuclei via desorption of the hydrocarbon species, and the growth of the SiC nuclei was disturbed the species. When the SiC nuclei were completely covered by graphene layers, which formed via a rearrangement of the excess solid C, they began to thermally decompose. Through the heating and cooling in the HTCVD process, the SiC nuclei embedded in the MHNs completely disappeared due to the size effect. This process enabled the MHNs to form, and the production of MHNs without SiC nuclei/crystals occurred at 2100 °C. The one-step growth of MHNs was possible due to the sequential formation and elimination of the SiC templates, the high temperature process, and the supply of hydrocarbons. The growth mechanism of the MHNs was established by comparing the experimental results and the thermodynamic simulations.

## Methods

### Preparation of MHNs using the HTCVD process

The HTCVD system consists of a vertical-type tube furnace with a quartz tube, which is surrounded by the cooling water and induction coils (Fig. S1). The induction heating was adjusted in the HTCVD process to control the growth temperature with graphite susceptors. More detailed information about the HTCVD is given in our previous reports^33,37,38,47^. TMS (99.9%) (Sigma-Aldrich Co.) and H_2_ (99.999%) were used as a source precursor and a carrier gas, respectively. The flow ratio of H_2_/TMS was fixed at 320, which was controlled by a mass flow controller (MFC). The working pressure, growth temperatures and growth time were 550 Torr, 1500 °C~2100 °C, and 1 hour, respectively. After the growth process, the temperature slowly cooled to room temperature.

### Characterization

The structural properties of the samples were analyzed by X-ray diffraction (XRD) (D/MAX-2500/PC, Rigaku), micro-Raman spectroscopy (LabRAM ARAMIS, Horiba Jobin-Yvon) and transmission electron microscopy (TEM) (JEOL-2100F, JEOL) techniques. The Cu-Kα radiation (λ = 0.154056 nm) with the 2 theta mode and 514.5 nm Ar^+^ ion laser was adjusted for the XRD and Raman instruments, respectively. The morphological and compositional analyses of all the samples were performed by both TEM and field emission scanning electron microscopy (FESEM) (JSM-7001F, JEOL) equipped with energy dispersive X-ray spectroscopy (EDS). Samples were fixed on the holder with carbon tape, and the FESEM analysis was carried out. For the TEM analysis preparation, the samples were mixed with ethanol and dispersed onto Cu grids to evenly disperse the samples. The thermodynamic calculations were performed using FactSage™ 6.4 software with the FactPS database.

## Electronic supplementary material

Supporting Information

## References

[1] Iijima S (1991). Helical microtubules of graphitic carbon. Nature.

[2] Novoselov KS (2004). Electric field effect in atomically thin carbon films. Science.

[3] Xu J, Zhang R, Wang J, Ge S, Wen F (2012). Hollow carbon onions with larger lattice spacing obtained by chlorination of the ball-milled SiC. Mater. Lett..

[4] Gorelik T, Urban S, Falk F, Kaiser U, Glatzed U (2003). Carbon onions produced by laser irradiation of amorphous silicon carbide. Chem. Phys. Lett..

[5] Pech D (2010). Ultrahigh-power micrometre-sized supercapacitors based on onion-like carbon. Nat. Nanotechnol..

[6] Han F-D, Yao B, Bai Y-J (2011). Preparation of carbon nano-onions and their application as anode materials for rechargeable lithium-ion batteries. J. Phys. Chem. C..

[7] Wei Z (2010). Nanoscale tunable reduction of graphene oxide for graphene electronics. Science.

[8] Dikin DA (2007). Preparation and characterization of graphene oxide paper. Nature.

[9] Etacheri V, Wang C, Connell MJ, Chan CK, Pol VG (2015). Porous carbon sphere anodes for enhanced lithium-ion storage. J. Mater. Chem. A.

[10] Asaka K, Terada T, Saito Y (2014). Transformation of silicon nanoparticles on a carbon nanotube heater into hollow graphitic nanocapsules via silicon carbide. Diam. Relat. Mater..

[11] Wang Y (2014). Electron beam “ballooned” carbon sphere derived from graphene oxide by a hydrazine assisted hydrothermal method. RSC Adv.

[12] Ugarte D (1993). Formation mechanism of quasi-spherical carbon particles induced by electron bombardment. Chem. Phys. Lett..

[13] Yang S, Zeng H, Zhao H, Zhang H, Cai W (2011). Luminescent hollow carbon shells and fullerene-like carbon spheres produced by laser ablation with toluene. J. Mater. Chem..

[14] Yoon S-M (2012). Synthesis of multilayer graphene balls by carbon segregation from nickel nanoparticles. ACS Nano.

[15] Tang K (2012). Hollow carbon nanospheres with superior rate capability for sodium-based batteries. Adv. Energy Mater..

[16] Kang J, Li OL, Saito N (2013). Synthesis of structure-controlled carbon nano spheres by solution plasma process. Carbon.

[17] Yen W-C (2014). Direct growth of self-crystallized graphene and graphite nanoballs with Ni vapor-assisted growth: From controllable growth to material characterization. Sci. Rep..

[18] Cambaz ZG, Yushin GN, Gogotsi Y (2006). Formation of carbide-derived carbon on β-silicon carbide whiskers. J. Am. Ceram. Soc..

[19] Zhang J, Kong L-B, Wang B, Luo Y-C, Kang L (2009). *In-situ* electrochemical polymerization of multi-walled carbon nanotube/polyaniline composite films for electrochemical supercapacitors. Synthetic Met..

[20] Zhang H (2008). Growth of manganese oxide nanoflowers on vertically-aligned carbon nanotube arrays for high-rate electrochemical capacitive energy storage. Nano Lett..

[21] Yang SJ (2009). Preparation and enhanced hydrostability and hydrogen storage capacity of CNT@MOF-5 hybrid composite. Chem. Mater..

[22] Yang Z (2013). Photovoltaic wire derived from a graphene composite fiber achieving an 8.45% energy conversion efficiency. Angew. Chem..

[23] Zhou M (2013). Highly conductive porous graphene/ceramic composites for heat transfer and thermal energy storage. Adv. Funct. Mater..

[24] Lightcap IV, Kamat PV (2013). Graphitic design: Prospects of graphene-based nanocomposites for solar energy conversion, storage, and sensing. Acc. Chem. Res..

[25] Seo SG (2014). Temperature-dependent charge transport in TiO_2_–multiwalled carbon nanotube composites. Carbon.

[26] Kim KT (2013). The influence of CNTs on the thermoelectric properties of a CNT/Bi_2_Te_3_ composite. Carbon.

[27] Pérez CR (2013). Structure and electrochemical performance of carbide-derived carbon nanopowders. Adv. Funct. Mater..

[28] Liu J, Wickramaratne NP, Qiao SZ, Jaroniec M (2015). Molecular-based design and emerging applications of nanoporous carbon spheres. Nat. Mater..

[29] Hoffman EN, Yushin G, Barsoum MW, Gogotsi Y (2005). Synthesis of carbide-derived carbon by chlorination of Ti_2_AlC. Chem. Mater..

[30] Emtsev KV (2009). Towards wafer-size graphene layers by atmospheric pressure graphitization of silicon carbide. Nat. Mater..

[31] Kusunoki M, Rokkaku M, Suzuki T (1997). Epitaxial carbon nanotube film self-organized by sublimation decomposition of silicon carbide. Appl. Phys. Lett..

[32] Kusunoki M, Suzuki T, Kaneko K, Ito M (1999). Formation of self-aligned carbon nanotube films by surface decomposition of silicon carbide. Phil. Mag. Lett..

[33] Nam D-H (2014). High-temperature chemical vapor deposition for SiC single crystal bulk growth using tetramethylsilane as a precursor. Cryst. Growth Des..

[34] Yakimova R, Janzen E (2000). Current status and advances in the growth of SiC. Diam. Relat. Mater..

[35] Ellison A (2004). SiC crystal growth by HTCVD. Mater. Sci. Forum.

[36] Ellison A (1999). High temperature CVD growth of SiC. Mater Sci Eng. B..

[37] Jeong S-M (2014). Synthesis of α-SiC from tetramethylsilane by chemical vapor deposition at high temperature. Appl. Phys. Express.

[38] Kim BG (2015). Condensation of vapor species at the outlets in high temperature chemical vapor deposition using tetramethylsilane as a precursor for SiC bulk growth. CrystEngComm.

[39] He CN, Shi CS, Du XW, Li JJ, Zhao NQ (2008). TEM investigation on the initial stage growth of carbon onions synthesized by CVD. J. Alloys Compd..

[40] Peng T, Lv H, He D, Pan M, Mu S (2013). Direct transformation of amorphous silicon carbide into graphene under low temperature and ambient pressure. Sci. Rep..

[41] Pimenta MA (2007). Studying disorder in graphite-based systems by Raman spectroscopy. Phys. Chem. Chem. Phys..

[42] Wasyluk J (2010). Raman investigation of different polytypes in SiC thin films grown by solid-gas phase epitaxy on Si (111) and 6H-SiC substrates. Mater. Sci. Forum.

[43] Norimatsu W, Kusunoki M (2009). Transitional structures of the interface between graphene and 6H–SiC (0001). Chem. Phys. Lett..

[44] Robinson J (2010). Nucleation of epitaxial graphene on SiC(0001). ACS Nano.

[45] Saito Y (1995). Nanoparticles and filled nanocapsules. Carbon.

[46] Michon A (2010). Direct growth of few-layer graphene on 6H-SiC and 3C-SiC/Si via propane chemical vapor deposition. Appl. Phys. Lett..

[47] Yoon J-Y (2016). Design of a high temperature chemical vapor deposition reactor in which the effect of the condensation of exhaust gas in the outlet is minimized using computational modeling. J. Cryst. Growth.

[48] Buffat PH, Borel J-P (1976). Size effect on the melting temperature of gold particles. Phys. Rev. A.

[49] Lai SL, Guo JY, Petrova V, Ramanath G, Allen LH (1996). Size-dependent melting properties of small tin particles: Nanocalorimetric measurements. Phys. Rev. Lett..

[50] Guisbiers G (2014). Gold−copper nano-alloy, “tumbaga”, in the era of nano: phase diagram and segregation. Nano Lett..

[51] Lu HM, Han FQ, Meng XK (2008). Size-dependent thermodynamic properties of metallic nanowires. J. Phys. Chem. B.

[52] Kim BG (2015). Facile fabrication of silicon and aluminum oxide nanotubes using antimony telluride nanowires as templates. Ceram. Int..

[53] Yim JWL, Xiang B, Wu J (2009). Sublimation of GeTe nanowires and evidence of its size effect studied by *in situ* TEM. J. Am. Chem. Soc..

