# Supplementary material for: One-step growth of multilayer-graphene hollow nanospheres via the self-elimination of SiC nuclei templates
Source: Sci Rep. 2017 Oct 23;7:13774. doi: 10.1038/s41598-017-13143-3 (PMC5653782; doi:10.1038/s41598-017-13143-3)
Supplement: Supplementary file 1 — Supporting Information [file 41598_2017_13143_MOESM1_ESM.doc]

Supporting Information for

**One-step growth of multilayer-graphene hollow nanospheres via the self-elimination of SiC nuclei templates**

By Byeong Geun Kim^1,2^, Deok-Hui Nam^2^, Seong-Min Jeong^2,*^, Myung-Hyun Lee^2^, Won-Seon Seo^2^,and Soon-Mok Choi^1,*^

^1^School of Energy, Materials and Chemical Engineering, Korea University of Technology and Education, Cheonan 31253, Korea

^2^Energy & Environmental Division, Korea Institute of Ceramic Engineering and Technology, Jinju 52851, Korea


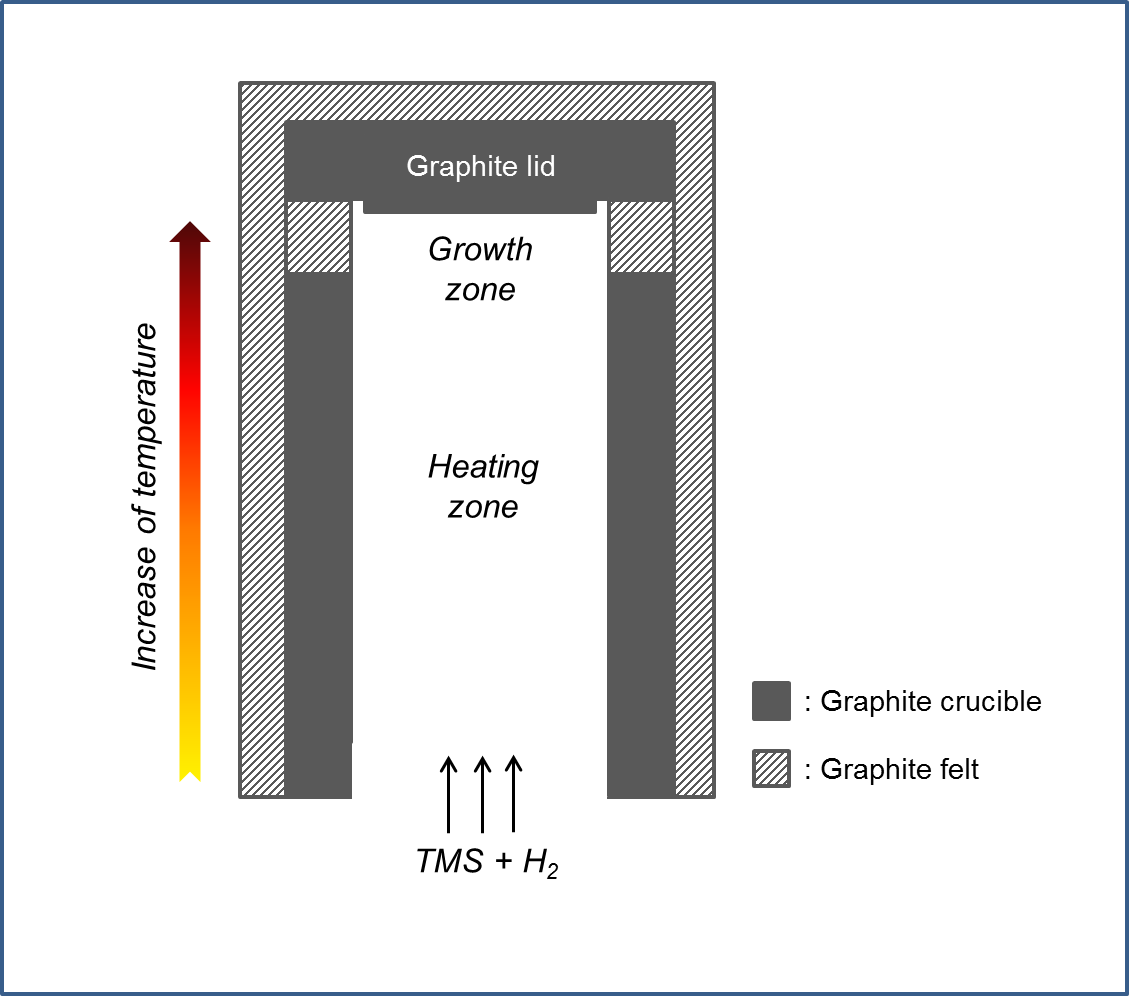


**Figure S1.** Schematic showing a cross section of the TMS-based HTCVD system. Graphite lid was used as a substrate, and the products formed on it.


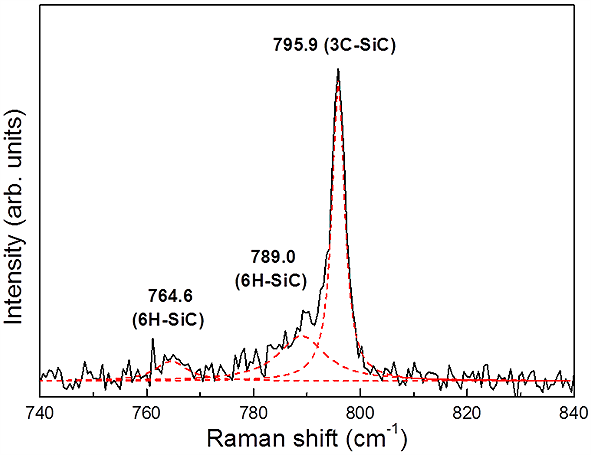


**Figure S2.** Raman spectrum of the SiC crystals obtained at 1900 °C. The dashed lines (red) are the Lorentzian fit to the experimental lines.


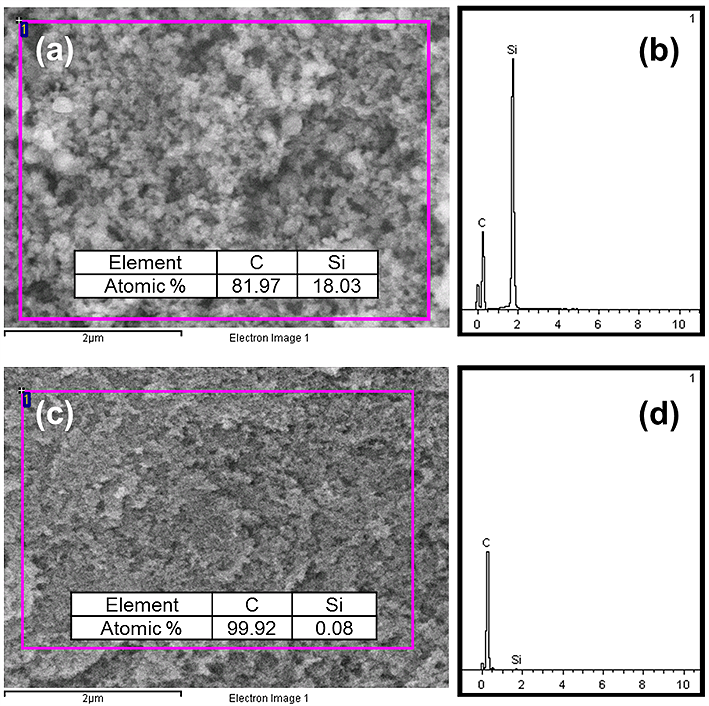


**Figure S3.** FESEM images and EDS spectra of the products obtained at (a)–(b) 1900 °C and (c)–(d) 2100 °C. EDS analyses were performed on the areas shown in pink solid lines. It is supposed that the very small content (0.08 at.%) of Si in Figs. S3(c) and S3(d) were contaminants in analysis equipment.


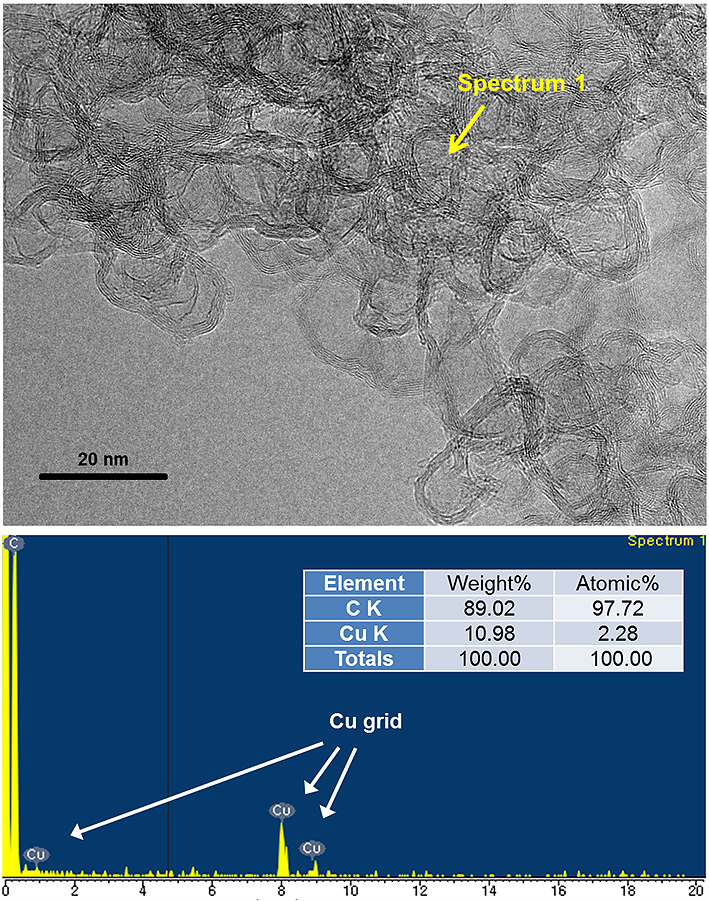


**Figure S4.** TEM image and EDS spectrum of the products (MHNs-2100) obtained at 2100 °C. The copper peaks in the EDS spectrum were originated from a copper grid, which was used to support the MHNs-2100.


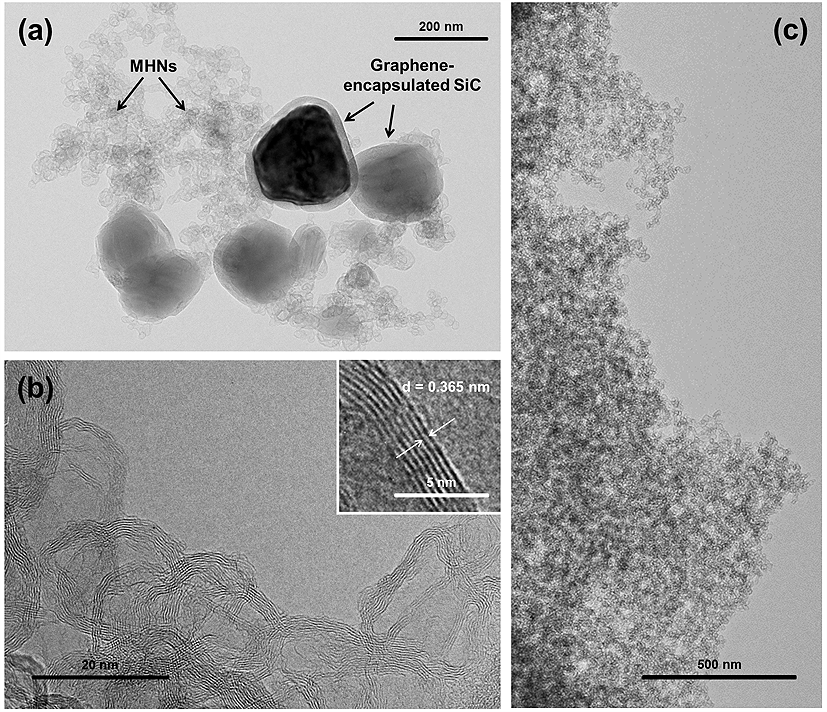


**Figure S5.** TEM images of the product obtained at a temperature of (a)–(b) 1900 °C and (c) 2100 °C.


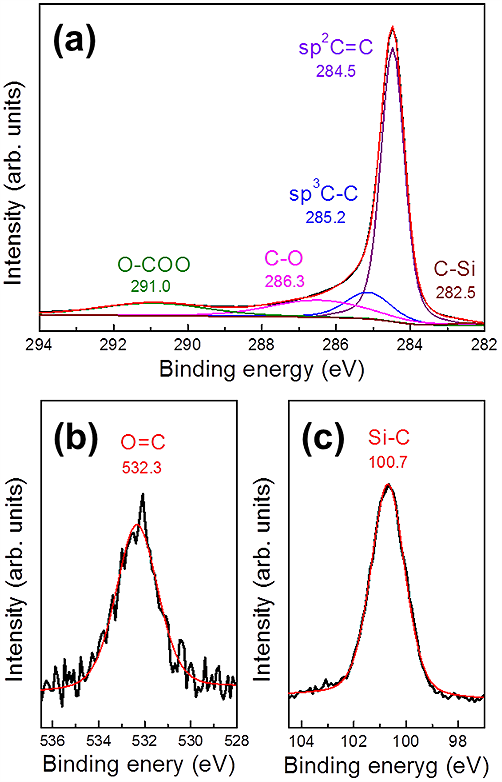


**Figure S6.** XPS spectra of (a) C, (b) O, and (c) Si in the product obtained at 1900 °C.

The binding energies of C, Si, and O were consisted with those of the previous reports [11,19,23]. The small content of O (0.64 at. %) was detected by XPS analyses, which may be attributed to a native oxidation by exposure to air after an experiment.


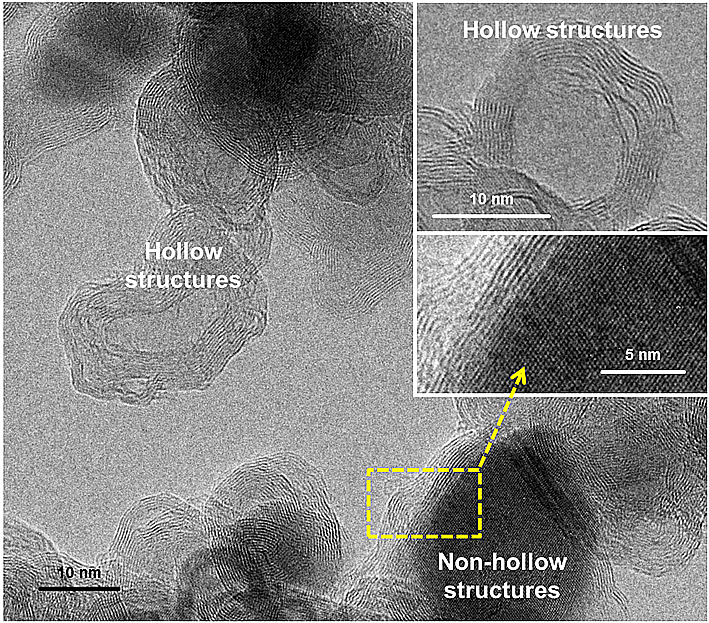


**Figure S7.** TEM images of the products acquired at 1500 °C.


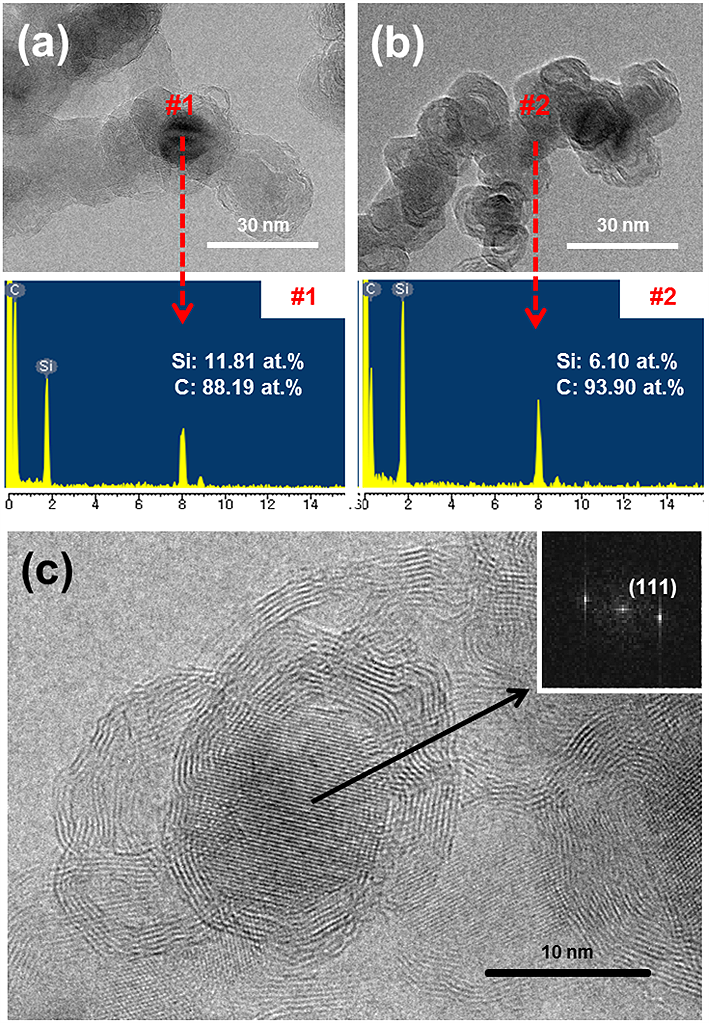


**Figure S8.** TEM images and EDS spectra of graphene-encapsulated SiC nuclei. Inset of Fig. 8(c) is the FFT image showing 3C-SiC crystalline nature.


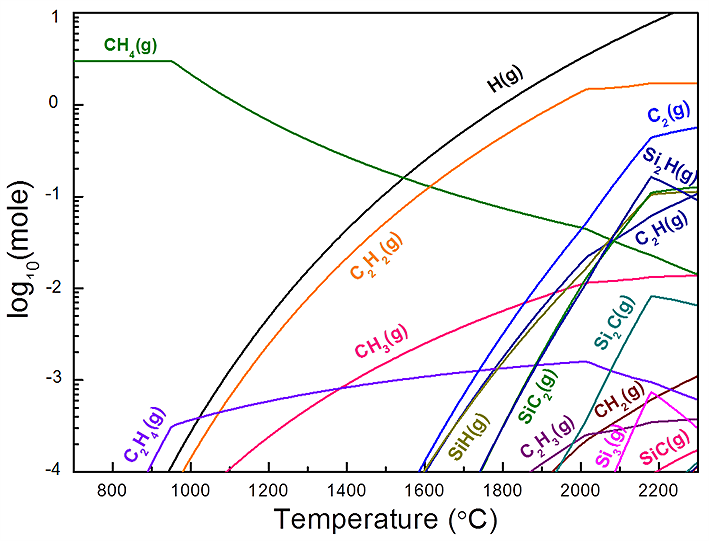


**Figure S9.** Evolution of thermodynamic equilibrium of gas species as a function of temperatures in TMS-based HTCVD process.


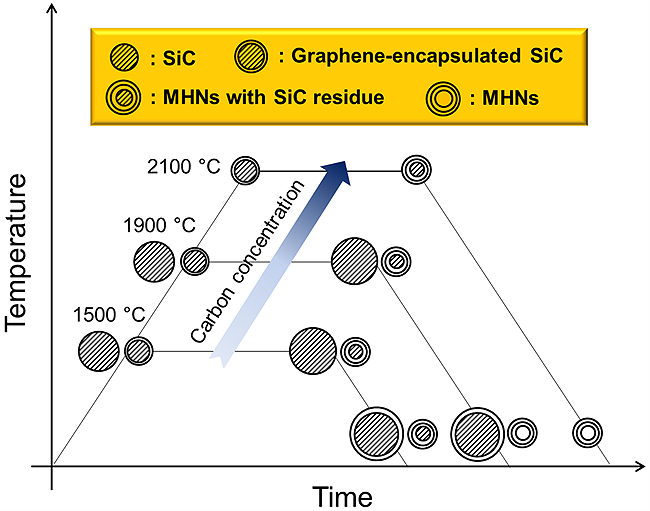


**Figure S10.** Schematic diagram showing the products formed with as a function of temperature, time, and carbon concentration and time.
